# Copeptin and risk stratification in patients with acute dyspnea

**DOI:** 10.1186/cc9336

**Published:** 2010-11-24

**Authors:** Mihael Potocki, Tobias Breidthardt, Alexandra Mueller, Tobias Reichlin, Thenral Socrates, Nisha Arenja, Miriam Reiter, Nils G Morgenthaler, Andreas Bergmann, Markus Noveanu, Peter T Buser, Christian Mueller

**Affiliations:** 1 Department of Cardiology, University Hospital, Petersgraben 4, Basel, 4031, Switzerland; 2 Department of Internal Medicine, University Hospital, Petersgraben 4, Basel, 4031, Switzerland; 3 Research Department, B.R.A.H.M.S. AG, Neuendorfstrasse 25, Hennigsdorf/Berlin, 16761, Germany

## Abstract

**Introduction:**

The identification of patients at highest risk for adverse outcome who are presenting with acute dyspnea to the emergency department remains a challenge. This study investigates the prognostic value of Copeptin, the C-terminal part of the vasopressin prohormone alone and combined to N-terminal pro B-type natriuretic peptide (NT-proBNP) in patients with acute dyspnea.

**Methods:**

We conducted a prospective, observational cohort study in the emergency department of a university hospital and enrolled 287 patients with acute dyspnea.

**Results:**

Copeptin levels were elevated in non-survivors (n = 29) compared to survivors at 30 days (108 pmol/l, interquartile range (IQR) 37 to 197 pmol/l) vs. 18 pmol/l, IQR 7 to 43 pmol/l; *P *< 0.0001). The areas under the receiver operating characteristic curve (AUC) to predict 30-day mortality were 0.83 (95% confidence interval (CI) 0.76 to 0.90), 0.76 (95% CI 0.67 to 0.84) and 0.63 (95% CI 0.53 to 0.74) for Copeptin, NT-proBNP and BNP, respectively (Copeptin vs. NTproBNP *P *= 0.21; Copeptin vs. BNP *P *= 0.002). When adjusted for common cardiovascular risk factors and NT-proBNP, Copeptin was the strongest independent predictor for short-term mortality in all patients (HR 3.88 (1.94 to 7.77); *P *< 0.001) and especially in patients with acute decompensated heart failure (ADHF) (HR 5.99 (2.55 to 14.07); *P *< 0.0001). With the inclusion of Copeptin to the adjusted model including NTproBNP, the net reclassification improvement (NRI) was 0.37 (*P *< 0.001). An additional 30% of those who experienced events were reclassified as high risk, and an additional 26% without events were reclassified as low risk.

**Conclusions:**

Copeptin is a new promising prognostic marker for short-term mortality independently and additive to natriuretic peptide levels in patients with acute dyspnea.

## Introduction

Acute dyspnea is a frequent clinical presentation in the emergency department (ED). Cardiac and pulmonary disorders account for more than 75% of patients presenting with acute dyspnea to the ED [1,2]. The identification of acute dyspneic patients at highest risk for death, particularly regarding short-term mortality remains a challenge. Patient history and physical examination remain the cornerstone of clinical evaluation [3], while disease specific scoring tools [4,5] and biomarkers such as natriuretic peptides have been introduced to assist the clinician in the diagnostic and prognostic assessment [6-10].

The arginin-vasopressin system plays a crucial role in the regulation of the individual endogenous stress response [11]. Levels of arginin-vasopressin have been shown to be elevated in heart failure [12] and in different states of shock [13], but investigation of the vasopressin system was limited so far due to the fact that vasopressin is unstable (half-life 5 to 15 minutes) and largely attached to platelets [14,15]. Copeptin, the c-terminal part of the vasopressin prohormone, is secreted stoichiometrically with vasopressin from the neurohypophysis and is much more stable, thus overcoming the limitations and difficulties assessing the arginin-vasopressin-system [16]. Recently, several studies investigated the prognostic role of Copeptin in various diseases [17-23], but little is known about the prognostic value of Copeptin in a typical ED population, for example, the patient group admitted with acute dyspnea. In clinical practice, the identification of dyspneic patients at highest risk for adverse outcome remains challenging. Therefore, we tested the prognostic value of Copeptin together with established markers such as BNP and NT-proBNP in an effort to better understand the role of Copeptin in this setting.

## Materials and methods

### Study population

The study population consisted of unselected patients presenting to the emergency department of the University Hospital of Basel, Switzerland, with a chief complaint of acute dyspnea. From April 2006 to March 2007, 292 patients (out of 327 patients screened) were prospectively enrolled. Exclusion criteria were age younger than 18 years, an obvious traumatic cause of dyspnea and patients on haemodialysis. 287 of the 292 patients had complete copeptin data at presentation and were considered as the study population. The study was carried out according to the principles of the Declaration of Helsinki and approved by the local ethics committee. Written informed consent was obtained from all participating patients.

### Clinical evaluation and follow-up

Patients underwent an initial clinical assessment including clinical history, physical examination, electrocardiogram, pulse oximetry, blood tests including BNP, and chest X-ray. Echocardiography, pulmonary function tests and other diagnostic tests like CT-angiography were performed according to the treating physician. CT-angiography was the imaging modality of choice in patients with suspected pulmonary embolism. To assess the dyspnea severity we used the NYHA (New York Heart Association) classification with NYHA II as 'dyspnea while walking up a slight incline', III as 'dyspnea while walking on level ground' and IV as 'dyspnea at rest'.

Two independent internists blinded to Copeptin reviewed all medical records including BNP levels and independently classified the patient's primary diagnosis into seven categories: acute decompensated heart failure (ADHF), acute exacerbation of chronic obstructive pulmonary disease, pneumonia, acute complications of malignancy, acute pulmonary embolism, hyperventilation, and others. In the event of diagnostic disagreement among the internist reviewers, they were asked to meet to come to a common conclusion. In the event that they were unable to come to a common conclusion, a third-party internist adjudicator was asked to review the data and determine which diagnosis was the most accurate.

The endpoint of the present study was defined as 30-day all-cause mortality. Each patient was contacted for follow-up, via telephone, by a single trained researcher after 30 days. Regarding mortality data, referring physicians were contacted or the administrative databases of respective hometowns were reviewed, if necessary. Of note, one patient was lost to follow-up, so mortality analyses were performed in 286 patients.

### Laboratory measurements

Blood samples for determination of Copeptin, BNP and NT-proBNP were collected at presentation into tubes containing potassium EDTA. After centrifugation, samples were frozen at -80°C until assayed in a blinded fashion in a single batch using a novel commercial sandwich immunoluminometric assay (B.R.A.H.M.S LUMItest CT-proAVP, BRAHMS AG, Hennigsdorf/Berlin, Germany) as described in detail elsewhere [16]. Since this initial publication, the assay was modified as follows: The capture antibody was replaced by a murine monoclonal antibody directed to amino acids 137 to 144 (GPAGAL) of pro-Arginin-Vasopressin. This modification improved the sensitivity of the assay. The lower detection limit was 0.4 pmol/L and the functional assay sensitivity (< 20% inter assay CV) was <1 pmol/L. Median Copeptin levels in 200 healthy individuals was 3.7 pmol/l and the 97.5^th ^percentile was 16.4 pmol/L. NT-proBNP levels were determined in a blinded fashion by a quantitative electrochemiluminescence immunoassay with CVs claimed by the manufacturer were 1.8% to 2.7% and 2.35% to 3.2% for within-run and total imprecision, respectively (Elecsys proBNP, Roche Diagnostics AG, Zug, Switzerland) [24] and BNP was measured by a microparticle enzyme immunoassay at the hospital laboratory with a CVs claimed by the manufacturer of 4.3% to 6.3% and 6.5% to 9.4% for within-run and total imprecision, respectively. (AxSym, Abbott Laboratories, Abbott Park, IL, USA) [25].

### Statistical analysis

Continuous variables are presented as mean ±SD or median (with interquartile range), and categorical variables as numbers and percentages. Univariate data on demographic and clinical features were compared by Mann-Whitney U-test or Fisher's exact test as appropriate. Correlations among continuous variables were assessed by the Spearman rank-correlation coefficient. Plasma levels of Copeptin, NT-proBNP and BNP were log-transformed to achieve a normal distribution. Thus, hazard ratios refer to a 10-fold rise in the levels of these markers. Receiver operating characteristic (ROC) curves were utilized to evaluate the accuracy of Copeptin, NT-proBNP and BNP to predict death. Areas under the curve (AUCs) were calculated for all markers. AUCs were compared according to the method by Hanley and McNeil [26]. We calculated the Net reclassification improvement (NRI) per Pencina *et al. *[27]. This index sums up the difference in proportions of patients that are reclassified to a higher or lower risk group predicted in terms of their actual outcome by adding Copeptin to the existing model with NT-proBNP and confounders. Cox regression analysis was assessed by univariate and multivariate analysis to identify independent predictors of outcome. In multivariate analysis, each of the three biomarkers (BNP, NT-proBNP and Copeptin) was included to the adjusted model including age, gender, a history of heart failure, glomerular filtration rate, diabetes and systolic blood pressure. In the next step, NT-proBNP or Copeptin was added to the adjusted model as dependent variable to examine the independent value of each biomarker. The Kaplan-Meier cumulative survival curves were compared by the log-rank test. Glomerular filtration rate was calculated using the abbreviated Modification of Diet in Renal Disease (MDRD) formula. Data were statistically analysed with SPSS 15.0 software (SPSS Inc., Chicago, IL, USA) and the MedCalc 9.3.9.0 package (MedCalc Software, Mariakerke, Belgium). All probabilities were two tailed and *P *< 0.05 was regarded as significant.

## Results

### Patient characteristics

The baseline characteristics of the 287 patients presenting with acute dyspnea are described in Table 1. Overall, mean age was 74 ± 12 years (median 77 years, interquartile range (IQR) 68 to 83 years), 52% were men and 80% were in NYHA functional class III and IV. The primary diagnosis was ADHF in 154 (54%) patients, acute exacerbation of chronic obstructive pulmonary disease in 57 (20%) patients, pneumonia in 32 (11%) patients, acute pulmonary embolism in 8 (3%) patients, acute complications of malignancy in 7 (2%) patients, hyperventilation in 5 (2%) patients, and other causes such as interstitial lung disease, asthma, or bronchitis in 24 (8%) patients. Differences between patients with ADHF versus patients without ADHF are depicted in Table 1.

**Table 1:** Baseline characteristics divided in patients with and without acute decompensated heart failure (ADHF)

Characteristic	Total (*n *= 287)	ADHF (*n *= 154)	No ADHF (*n *= 133)	*P*-value
Age (years)*^a^*	74 ± 12	78 ± 9	68 ± 13	< 0.0001
Male sex (% of patients)	52	51	53	0.906
BMI (kg/m^2^)*^a^*	26.1 ± 6.2	26.6 ± 5.9	25.5 ± 6.5	0.124
*Medical conditions (% of patients)*				
Heart failure	24	40	7	< 0.0001
Coronary artery disease	28	38	16	< 0.0001
Chronic obstructive pulmonary disease	34	27	42	0.006
Diabetes	18	24	11	0.005
Hypertension	68	78	56	< 0.0001
Hyperlipidemia	29	33	25	0.165
Chronic kidney disease	28	44	11	< 0.0001
*Initial clinical findings*				
Heart rate (bpm)*^a^*	93 ± 23	93 ± 25	92 ± 19	0.495
Systolic pressure (mm Hg)*^a^*	138 ± 26	135 ± 27	140 ± 25	0.098
NYHA functional class (% of patients)				
II	20	10	32	< 0.0001
III	40	45	35	0.109
IV	40	45	33	0.034
Edema	42	57	26	< 0.0001
Rales	54	64	43	< 0.0001
*Medication at admission*				
Beta-blockers	39	57	17	< 0.0001
ACE-Inhibitors/AT-receptor-blockers	49	62	34	< 0.0001
Diuretics	52	64	39	< 0.0001
*Laboratory findings*				
eGFR - ml/min/1.73m2 *^b^*	67 (44 to 89)	54 (36 to 73)	80 (63 to 112)	< 0.0001
BNP (pmol/l) *^b^*	349 (89 to 1,121)	976 (467 to 1,925)	81 (39 to 181)	< 0.0001
NT-proBNP (pmol/l) *^b^*	1,656 (314 to 6,105)	5,757 (1,924 to 13,243)	300 (76 to 974)	< 0.0001
Copeptin (pmol/l) *^b^*	21 (8 to 52)	34 (13 to 71)	11 (6 to 31)	< 0.0001

*^a ^*mean ± plusorminus SD, *^b ^*median (IQR = interquartile range).

BMI, body mass index; eGRF, estimated glomerular filtration rate; NYHA, New York Heart Association; BNP, B-type natriuretic peptide; NT-proBNP, N-terminal pro-B-type natriuretic peptide.

### Copeptin levels and prognostic value of Copeptin on short-term outcome

The median Copeptin concentration was 21 pmol/l (IQR 8 to 52 pmol/l) in all patients. Concentrations of Copeptin were significantly higher in those dyspneic patients with ADHF versus those without (34 pmol/l, IQR 13 to 71 pmol/l vs.11 pmol/l, IQR 6 to 31 pmol/l; *P *< 0.0001). There was only a modest correlation between concentrations of log-transformed Copeptin and BNP (r = 0.42, *P *< 0.001) or NT-proBNP (r = 0.53, *P *< 0.001).

**Figure 1:**
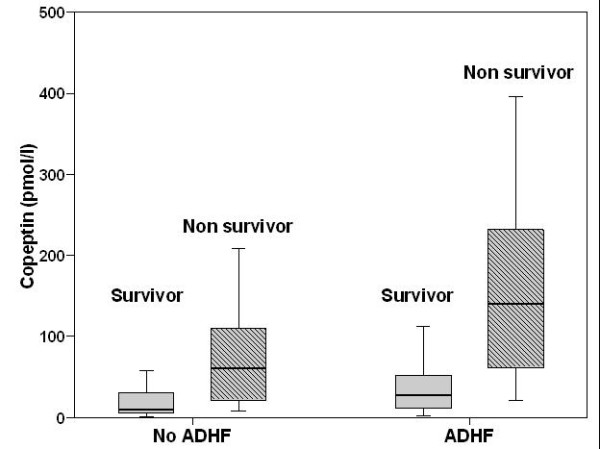
**Copeptin levels according to survival in patients with and without acute decompensated heart failure (ADHF)**.

At 30 days, 29 patients (10.1%) had died. Non-survivors had significantly higher Copeptin levels than survivors (108 pmol/l, IQR 37 to 197 pmol/l) vs. 18 pmol/l, IQR 7 to 43 pmol/l; *P *< 0.0001). Among those subjects with ADHF, non-survivors (n = 21) had higher Copeptin levels than survivors (n = 133) (140 pmol/l, IQR 60 to 236 pmol/l vs. 28 pmol/l, IQR 11 to 55 pmol/l; *P *< 0.0001) and also in patients without ADHF the levels of Copeptin were higher in non-survivors (n = 8) than in survivors (n = 125) (61 pmol/l, IQR 18 to 112 pmol/l vs. 10 pmol/l, IQR 6 to 30 pmol/l; *P *= 0.007; Figure 1).

**Figure 2:**
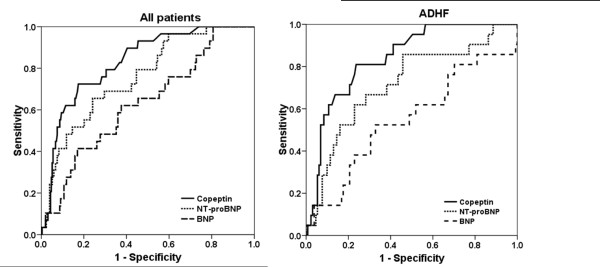
**Diagnostic accuracy for for Copeptin, NT-proBNP and BNP to predict 30-day mortality**. AUC: Area under the receiver operating characteristic curve; NT-proBNP: N-terminal pro B-type natriuretic peptide; BNP: B-type natriuretic peptide; ADHF: acute decompensated heart failure

In Figure 2 the AUC to predict mortality are illustrated for Copeptin, NT-proBNP and BNP. The ROC analyses demonstrated an AUC of 0.83 (95% confidence interval (CI) 0.76 to 0.90) for Copeptin to predict 30-day mortality, with an optimal cut-point of 59 pmol/l. NT-proBNP had an AUC of 0.76 (95% CI 0.67 to 0.84) and BNP of 0.63 (95% CI 0.53 to 0.74) for 30-day mortality. Copeptin had a significantly higher AUC compared with BNP (*P *= 0.002) but not compared to NT-proBNP (*P *= 0.21). In patients with ADHF the AUC were 0.84 (95% CI 0.76 to 0.92) for Copeptin, 0.72 (95% CI 0.59 to 0.84) for NT-proBNP and 0.55 (95% CI 0.40 to 0.69) for BNP (Copeptin vs. NT-proBNP *P *= 0.098; Copeptin vs. BNP *P *< 0.001).

**Figure 3:**
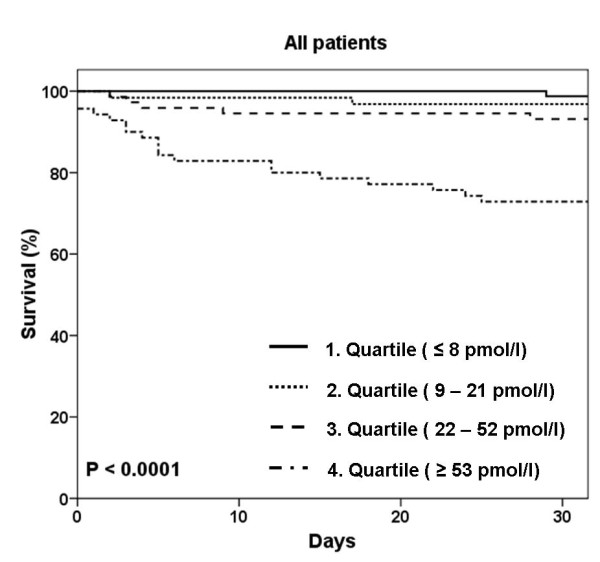
**Kaplan-Meier curves demonstrating survival over time according to quartiles of Copeptin at baseline in all patients**.

Kaplan-Meier curves showed that patients in the highest quartile (Q4) had a significantly increased mortality compared with the other quartiles (Q1 to Q3) (Q1 = 1.2%, Q2 = 3.2%, Q3 = 6.8%, Q4 = 30%; *P *< 0.0001) (Figure 3). This pattern of increased mortality according to quartiles remained true for patients with ADHF but not for patients without ADHF (*P *< 0.0001 and *P *= 0.121, respectively). Patients in the highest quartile more often required admission to the hospital (99% vs. 83% in Q1 to Q3; *P *< 0.001), more often required admission to the intensive care unit (ICU) (16% vs. 6% Q1 to Q3, *P *= 0.011) and had higher in-hospital mortality (19% vs. 3% in Q1 to Q3, *P *< 0.0001).

Univariate Cox regression analysis showed that plasma levels of Copeptin, NT-proBNP, glomerular filtration rate and systolic blood pressure were predictors of 30-day mortality in all patients, regardless of whether they had ADHF or not. Copeptin was the strongest predictor of mortality in all patients (HR 5.28, 95% CI 3.08 to 9.06, *P *< 0.0001) and in patients with ADHF (HR 5.36, 95% CI 2.82 to 10.19, *P *< 0.001). BNP showed only a limited prognostic value in all patients and no value in the subgroups (Table 2).

**Table 2:** Univariate Cox regression analysis for 30-day mortality

	All patients		ADHF		No ADHF	
			
Characteristic	HR for 30-day mortality	*P*-value	HR for 30-day mortality	*P*-value	HR for 30-day mortality	*P*-value
Age (yr)	1.05 (1.01 to 1.10)	0.012	1.03 (0.98 to 1.09)	0.230	1.06 (0.99 to 1.13)	0.1
Male sex	1.55 (0.73 to 3.27)	0.255	1.59 (0.66 to 3.84)	0.300	1.51 (0.36 to 6.33)	0.57
BMI	0.99 (0.93 to 1.05)	0.68	1.01 (0.94 to 1.08)	0.830	0.91 (0.80 to 1.04)	0.18
Medical conditions (% of patients)						
Heart failure	1.40 (0.64 to 3.08)	0.4	1.13 (0.48 to 2.69)	0.780	0.05 (0.00 to 7228)	0.61
Coronary artery disease	0.52 (0.20 to 1.35)	0.18	0.47 (0.17 to 1.29)	0.140	0.04 (0.00 to 127)	0.43
Chronic obstructive pulmonary disease	0.47 (0.33 to 1.68)	0.48	1.12 (0.44 to 2.89)	0.810	0.46 (0.09 to 2.26)	0.34
Diabetes	0.72 (0.25 to 2.06)	0.53	0.50 (0.15 to 1.70)	0.270	1.16 (0.14 to 9.40)	0.89
Hypertension	0.77 (0.36 to 1.62)	0.49	0.52 (0.21 to 1.28)	0.150	0.80 (0.20 to 3.20)	0.75
Hyperlipidemia	0.37 (0.13 to 1.06)	0.07	0.32 (0.10 to 1.09)	0.070	0.41 (0.05 to 3.34)	0.41
Chronic kidney disease	2.89 (1.39 to 5.98)	0.004	1.47 (0.62 to 3.46)	0.380	9.64 (2.41 to 38.61)	0.001
Initial clinical findings						
Heart rate (bpm)	1.01 (0.99 to 1.02)	0.45	1.00 (0.98 to 1.02)	0.870	1.02 (0.99 to 1.05)	0.22
Systolic pressure (mm Hg)	0.97 (0.96 to 0.99)	< 0.001	0.98 (0.96 to 0.99)	0.010	0.97 (0.94 to 1.00)	0.02
NYHA functional class	3.44 (1.74 to 6.81)	< 0.001	7.47 (2.28 to 24.45)	0.001	1.53 (0.65 to 3.67)	0.33
Edema	1.73 (0.83 to 3.59)	0.14	1.02 (0.43 to 2.42)	0.960	3.00 (0.75 to 12.00)	0.12
Rales	1.40 (0.66 to 2.95)	0.38	0.72 (0.30 to 1.74)	0.460	4.14 (0.84 to 20.53)	0.08
*Medication at admission*						
Beta-blockers	0.82 (0.38 to 1.76)	0.61	0.65 (0.28 to 1.53)	0.320	0.04 (0.00 to 90)	0.41
ACE-Inhibitors/AT-receptor-blockers	0.95 (0.46 to 1.96)	0.88	0.51(0.22 to 1.20)	0.120	1.96 (0.49 to 7.85)	0.34
Diuretics	1.76 (0.82 to 3.79)	0.15	1.40 (0.54 to 3.60)	0.490	1.62 (0.41 to 6.49)	0.49
*Laboratory findings*						
eGFR - ml/minute/1.73m2 *^a^*	0.11 (0.04 to 0.35)	< 0.001	0.18 (0.04 to 0.81)	0.025	0.08 (0.01 to 0.55)	0.011
BNP *^a^*	2.02 (1.14 to 3.58)	0.017	1.23 (0.44 to 3.41)	0.700	3.66 (0.83 to 16.11)	0.086
NT-proBNP *^a^*	3.64 (2.02 to 6.56)	< 0.0001	4.57 (1.72 to 12.17)	0.002	8.26 (2.34 to 29.12)	0.001
Copeptin *^a^*	5.28 (3.08 to 9.06)	< 0.0001	5.36 (2.82 to 10.19)	< 0.001	4.21 (1.46 to 12.14)	0.008

*^a ^*log-transformed to achieve normal distribution.

BMI, body mass index; eGRF, estimated glomerular filtration rate; NYHA, New York Heart Association; BNP, B-type natriuretic peptide; NT-proBNP, N-terminal pro-B-type natriuretic peptide.

Table 3 shows the multivariate Cox regression analysis after adjustment for common cardiovascular risk factors (age, gender, history of heart failure, glomerular filtration rate, diabetes, systolic blood pressure). Copeptin remained the strongest independent predictor for 30-day mortality in all patients (HR 4.58, 95% CI 2.29 to 9.13; *P *< 0.001), especially in a patient with ADHF (HR 6.51, 95% CI 2.83 to 14.95; *P *< 0.0001). Even when NT-proBNP was included in this model, Copeptin kept its prognostic value in all patients (HR 3.88, 95% CI 1.94 to 7.77; *P *= 0.0001) and even more in patients with ADHF (HR 5.99, 95% CI 2.55 to 14.07; *P *< 0.0001). NT-proBNP was not significantly associated with mortality in patients with ADHF after including Copeptin in the model (HR 2.78, 95% CI 0.78 to 10.60; *P *= 0.11). The addition of Copeptin to the adjusted model including NT-proBNP resulted in reclassification of 37% of the patients. The NRI for events was 0.21 and the NRI for non-events was 0.16, achieving an NRI for the entire study cohort of 0.37 at 30 days (*P *< 0.001). Overall, 13 patients (30%) of those who experienced events were reclassified as high risk, and an additional 64 patients (26%) without events were reclassified as low risk.

**Table 3:** Multivariate Cox regression analysis for 30-day mortality

	All patients		ADHF		No ADHF	
			
Variable	HR for 30-day mortality	*P*-value	HR for 30-day mortality	*P*-value	HR for 30-day mortality	*P*-value
Copeptin *^a^*	4.58 (2.29 to 9.13)	< 0.0001	6.51 (2.83 to 14.95)	< 0.0001	1.87 (0.29 to 12.12)	0.51
BNP *^a^*	1.42 (0.71 to 2.86)	0.32	0.76 (0.23 to 2.50)	0.65	2.28 (0.49 to 10.67)	0.3
NT-proBNP *^a^*	3.17 (1.49 to 6.71)	0.003	3.84 (1.15 to 12.89)	0.029	5.32 (1.1 to 25.76)	0.038
						
Adjusted model including NT to proBNP or Copeptin						
Copeptin *^a^*	3.88 (1.94 to 7.77)	< 0.001	5.99 (2.55 to 14.07)	< 0.0001	1.23 (0.17 to 9.14)	0.84
NT-proBNP *^a^*	2.74 (1.27 to 5.93)	0.01	2.78 (0.78 to 10.60)	0.11	5.26 (1.07 to 25.79)	0.041

Adjusted for age, gender, history of heart failure, glomerular filtration rate, diabetes, systolic blood pressure. *^a ^*log-transformed to achieve normal distribution. BNP, B-type natriuretic peptide; NT-proBNP, N-terminal pro-B-type natriuretic peptide; HR, hazard ratio.

### Combined prognostic value of Copeptin and NT-proBNP

**Figure 4:**
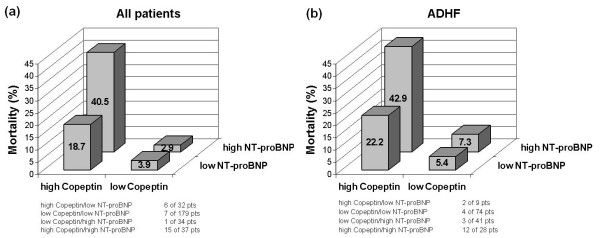
**30-day mortality as a function of Copeptin and NT-proBNP concentrations in all patients (a) and in patients with ADHF (b)**. NT-proBNP: N-terminal pro B-type natriuretic peptide; ADHF: acute decompensated heart failure.

Mortality rates in all dyspneic patients as a function of Copeptin and NT-proBNP concentrations were examined and showed in Figure 4. Those with low Copeptin concentrations (lowest three quartiles) had lower rates of death in the first 30 days, irrespective of NT-proBNP concentration. Those patients with elevations (highest quartile) in both, Copeptin and NT-proBNP had the highest rates of death (15 out of 37 patients, 40.5%) at 30 days. Considering those patients with ADHF, similar to the group as a whole, the same relationship between Copeptin, NT-proBNP and outcome was observed. Those patients with low rates of Copeptin had low rates of death, irrespective of NT-proBNP levels at 30 days. In those patients with an elevation of both, Copeptin and NT-proBNP, the mortality rate was 42.9%, with 12 deaths out of 28 patients at 30 days.

## Discussion

The early risk stratification of patients with acute dyspnea admitted to the ED is an unmet clinical need to improve the patient care in the first days of hospitalisation. Therefore, we investigated the role of Copeptin to predict short-term mortality in patients presenting with acute dyspnea to the ED. To the best of our knowledge, this is the first study analysing Copeptin plasma levels and their additive value to natriuretic peptides in a broader patient population like the typical ED population admitted with acute dyspnea.

We report five major findings. First, Copeptin levels were significantly higher in patients with ADHF than in patients with other diagnoses responsible for acute dyspnea. Second, Copeptin was significantly higher in non-survivors compared to survivors at 30 days, regardless of whether ADHF was present or not. Third, Copeptin is a new promising prognostic marker for short-term mortality independent of natriuretic peptide levels in patients with acute dyspnea and even more in patients with ADHF. Fourth, patients in the highest quartile of Copeptin levels had the highest mortality rate. Fifth, and of most importance, because both Copeptin and NT-proBNP were independent predictors of death, we also showed that the elevation of both markers was associated with the highest rates of death at 30 days in the entire patient cohort as well as in patients with ADHF. Furthermore, patients with low Copeptin levels had an excellent short-term prognosis even if NT-proBNP levels were high. These findings have major clinical implications. In clinical practice, the identification of dyspneic patients at highest risk for adverse outcomes remains difficult and largely depends on the underlying cause. Adding to this complexity is the fact that acute dyspnea is often due to multiple reasons like cardiac, pulmonary or inflammatory causes. Specific markers of the cardiovascular system like natriuretic peptides may therefore not be ideal to predict the outcome of patients with acute dyspnea in the ED.

Katan *et al*. showed in a small study that Copeptin is a good marker of the individual stress level comparing three groups of patients with increasing stress levels (healthy controls without apparent stress, hospitalized medical patients with moderate stress and surgical patients 30 minutes after extubation, with maximal stress) [28]. Taking this into account, it seems reasonable that patients with acute dyspnea and apparently in a stress situation could have higher Copeptin levels.

Recent studies by our group and others have suggested Copeptin and, therefore, the vasopressin system to be major determinants of outcome in patients where dyspnea is often the major symptom such as in acute myocardial infarction, in chronic heart failure and even in patients with community-acquired pneumonia and exacerbated chronic obstructive pulmonary disease [17-22,29].

Copeptin release in acutely dyspneic patients is most likely related to three possible mechanisms. First, vasopressin release in heart failure is mainly driven by arterial underfilling caused by cardiac output failure, which activates the baroreceptors in the carotid sinus and the aortic arch. Volume overload and hyponatremia might also stimulate the release of vasopressin [30,31]. Second, vasopressin has been shown to have vasoconstrictive effects, which may correlate to the hypoxia induced-vasoconstriction in severe chronic obstructive pulmonary disease [32,33]. Increased concentrations of vasopressin may compensate for vasopressin (V1) receptor down-regulation following exposure to sustained hypoxemia [34]. Third, Copeptin is significantly increased in bacterial infection and febrile conditions [13,35]. In the present study, the main causes of acute dyspnea were ADHF (54%), acute exacerbation of chronic obstructive pulmonary disease (20%) and pneumonia (11%). Therefore, the above mentioned mechanisms of Copeptin release reflect the broad spectrum of our acutely dyspneic patients and our findings corroborate that Copeptin may be a new promising candidate for the prediction of short-term mortality in this patient population. One could argue that Copeptin is another unspecific marker of inflammation. We, therefore, also compared the prognostic value of Copeptin with CRP and found that Copeptin was superior to CRP, an established inflammatory and also prognostic marker (AUC 0.83 (95% CI 0.76 to 0.90) vs. 0.71 (0.63 to 0.80), *P *= 0.04). Further studies should investigate whether Copeptin might help physicians tailor the therapy in view of the relative risk and allocate resources accordingly and whether this risk-stratification guided strategy might affect outcome. Additionally, it should be investigated whether serial copeptin testing further improves the risk stratification of acute dyspneic patients.

There are several limitations to our study. First, data derived from a single-centre study always need to be replicated in larger multicentre studies. However, our cohort is representative because patient characteristics are comparable to multicentre studies of acute dyspnea [1,36]. Second, we assessed all-cause mortality because classification of death in clinical practice can sometimes be difficult and unreliable [37]. However, exact numbers of all different causes of death could have provided more interesting insights into the pathophysiological role of the biomarkers. And finally, the diagnosis of ADHF remains challenging even with the use of BNP and the diagnostic classification was possibly not 100% accurate. It is possible that some patients have had latent or mild heart failure, even though they were classified in the 'no ADHF'-group because other information (for example, a history of chronic obstructive pulmonary disease, pulmonary infiltrates on chest x-ray) may have suggested that the diagnosis was not ADHF. However, our classification was obtained by experienced physicians, suggesting that our results are valid.

## Conclusions

Our study suggests that Copeptin alone or combined with NT-proBNP has a potential to assist clinicians in risk stratifying patients presenting with acute dyspnea regarding short-term mortality.

## Key messages

• In patients with acute dyspnea, Copeptin levels are elevated in non-survivors compared to survivors.

• Copeptin is a new promising prognostic marker for short-term mortality independently of natriuretic peptide levels.

• The elevation of both Copeptin and NT-proBNP was associated with the highest rates of death at 30 days.

• Patients with low Copeptin levels had an excellent short-term prognosis even if NT-proBNP levels were high.

## Abbreviations

ADHF: acute decompensated heart failure; AUC: area under the curve; BNP: B-type natriuretic peptide; CI: confidence interval; ED: emergency department; HR: hazard ratio; ICU: intensive care unit; IQR: interquartile range; NRI: net reclassification index; NT-proBNP: N-terminal pro-B-type natriuretic peptide; ROC: receiver operating characteristic curves.

## Competing interests

CM has received research support from Abbott, Biosite, Brahms, Roche, and Siemens as well as Behring. AB is an employee of BRAHMS AG, which is a company developing and marketing *in vitro *diagnostic products, including the Copeptin assay used in this manuscript. Also, AB holds patent applications related to this technology, and is a shareholder of BRAHMS AG. NM is an employee of BRAHMS AG. The other co-authors have no competing interests.

## Authors' contributions

MP and CM participated in study concept and design, acquisition of data, analysis and interpretation of data, drafting of the manuscript, critical revision of the manuscript for important intellectual content. They also had full access to all of the data in the study and take responsibility for the integrity of the data and the accuracy of the data analysis. TB, TR, MN, TS, AM, NA and MR participated in acquisition of data, analysis and interpretation of data and critical revision of the manuscript for important intellectual content. NM and AB participated in analysis and interpretation of data and critical revision of the manuscript for important intellectual content. PB participated in analysis, interpretation of data, drafting of the manuscript and critical revision of the manuscript for important intellectual content. All authors read and approved the final manuscript.

## References

[B1] Maisel AS Krishnaswamy P Nowak RM McCord J Hollander JE Duc P Omland T Storrow AB Abraham WT Wu AH Clopton P Steg PG Westheim A Knudsen CW Perez A Kazanegra R Herrmann HC McCullough PA Rapid measurement of B-type natriuretic peptide in the emergency diagnosis of heart failure N Engl J Med 2002 347 161 167 10.1056/NEJMoa020233 12124404

[B2] Mueller C Scholer A Laule-Kilian K Martina B Schindler C Buser P Pfisterer M Perruchoud AP Use of B-type natriuretic peptide in the evaluation and management of acute dyspnea N Engl J Med 2004 350 647 654 10.1056/NEJMoa031681 14960741

[B3] Dyspnea. Mechanisms, assessment, and management: a consensus statement. American Thoracic Society Am J Respir Crit Care Med 1999 159 321 340 9872857 10.1164/ajrccm.159.1.ats898

[B4] Fine MJ Auble TE Yealy DM Hanusa BH Weissfeld LA Singer DE Coley CM Marrie TJ Kapoor WN A prediction rule to identify low-risk patients with community-acquired pneumonia N Engl J Med 1997 336 243 250 10.1056/NEJM199701233360402 8995086

[B5] Fonarow GC Adams KF Jr Abraham WT Yancy CW Boscardin WJ Risk stratification for in-hospital mortality in acutely decompensated heart failure: classification and regression tree analysis JAMA 2005 293 572 580 10.1001/jama.293.5.572 15687312

[B6] Richards AM Nicholls MG Yandle TG Frampton C Espiner EA Turner JG Buttimore RC Lainchbury JG Elliott JM Ikram H Crozier IG Smyth DW Plasma N-terminal pro-brain natriuretic peptide and adrenomedullin: new neurohormonal predictors of left ventricular function and prognosis after myocardial infarction Circulation 1998 97 1921 1929 9609085 10.1161/01.cir.97.19.1921

[B7] Knudsen CW Clopton P Westheim A Klemsdal TO Wu AH Duc P McCord J Nowak RM Hollander JE Storrow AB Abraham WT McCullough PA Maisel AS Omland T Predictors of elevated B-type natriuretic peptide concentrations in dyspneic patients without heart failure: an analysis from the breathing not properly multinational study Ann Emerg Med 2005 45 573 580 10.1016/j.annemergmed.2005.01.027 15940086

[B8] Ruggiano G Camajori-Tedeschini R Lombardi V Pratesi M Rosselli A 287: Plasma BNP Levels in the Risk Stratification of Septic Patients at the Emergency Department Annals of Emergency Medicine 2008 51 557 558 10.1016/j.annemergmed.2008.01.258

[B9] Christ M Thuerlimann A Laule K Klima T Hochholzer W Perruchoud AP Mueller C Long-term prognostic value of B-type natriuretic peptide in cardiac and non-cardiac causes of acute dyspnoea Eur J Clin Invest 2007 37 834 841 10.1111/j.1365-2362.2007.01871.x 17931382

[B10] Gegenhuber A Mueller T Dieplinger B Poelz W Pacher R Haltmayer M B-type natriuretic peptide and amino terminal proBNP predict one-year mortality in short of breath patients independently of the baseline diagnosis of acute destabilized heart failure Clin Chim Acta 2006 370 174 179 10.1016/j.cca.2006.02.010 16600203

[B11] Itoi K Jiang YQ Iwasaki Y Watson SJ Regulatory mechanisms of corticotropin-releasing hormone and vasopressin gene expression in the hypothalamus J Neuroendocrinol 2004 16 348 355 10.1111/j.0953-8194.2004.01172.x 15089973

[B12] Goldsmith SR Gheorghiade M Vasopressin antagonism in heart failure J Am Coll Cardiol 2005 46 1785 1791 10.1016/j.jacc.2005.02.095 16286160

[B13] Jochberger S Mayr VD Luckner G Wenzel V Ulmer H Schmid S Knotzer H Pajk W Hasibeder W Friesenecker B Mayr AJ Dunser MW Serum vasopressin concentrations in critically ill patients Crit Care Med 2006 34 293 299 10.1097/01.CCM.0000198528.56397.4F 16424705

[B14] Robertson GL Mahr EA Athar S Sinha T Development and clinical application of a new method for the radioimmunoassay of arginine vasopressin in human plasma J Clin Invest 1973 52 2340 2352 10.1172/JCI107423 4727463 PMC333039

[B15] Preibisz JJ Sealey JE Laragh JH Cody RJ Weksler BB Plasma and platelet vasopressin in essential hypertension and congestive heart failure Hypertension 1983 5 I129 138 6826223 10.1161/01.hyp.5.2_pt_2.i129

[B16] Morgenthaler NG Struck J Alonso C Bergmann A Assay for the measurement of copeptin, a stable peptide derived from the precursor of vasopressin Clin Chem 2006 52 112 119 10.1373/clinchem.2005.060038 16269513

[B17] Voors AA von Haehling S Anker SD Hillege HL Struck J Hartmann O Bergmann A Squire I van Veldhuisen DJ Dickstein K C-terminal provasopressin (copeptin) is a strong prognostic marker in patients with heart failure after an acute myocardial infarction: results from the OPTIMAAL study Eur Heart J 2009 30 1187 1194 10.1093/eurheartj/ehp098 19346228

[B18] Masia M Papassotiriou J Morgenthaler NG Hernandez I Shum C Gutierrez F Midregional pro-A-type natriuretic peptide and carboxy-terminal provasopressin may predict prognosis in community-acquired pneumonia Clin Chem 2007 53 2193 2201 10.1373/clinchem.2007.085688 17951293

[B19] Neuhold S Huelsmann M Strunk G Stoiser B Struck J Morgenthaler NG Bergmann A Moertl D Berger R Pacher R Comparison of copeptin, B-type natriuretic peptide, and amino-terminal pro-B-type natriuretic peptide in patients with chronic heart failure: prediction of death at different stages of the disease J Am Coll Cardiol 2008 52 266 272 10.1016/j.jacc.2008.03.050 18634981

[B20] Stolz D Christ-Crain M Morgenthaler NG Leuppi J Miedinger D Bingisser R Muller C Struck J Muller B Tamm M Copeptin, C-reactive protein, and procalcitonin as prognostic biomarkers in acute exacerbation of COPD Chest 2007 131 1058 1067 10.1378/chest.06-2336 17426210

[B21] Khan SQ Dhillon OS O'Brien RJ Struck J Quinn PA Morgenthaler NG Squire IB Davies JE Bergmann A Ng LL C-terminal provasopressin (copeptin) as a novel and prognostic marker in acute myocardial infarction: Leicester Acute Myocardial Infarction Peptide (LAMP) study Circulation 2007 115 2103 2110 10.1161/CIRCULATIONAHA.106.685503 17420344

[B22] Gegenhuber A Struck J Dieplinger B Poelz W Pacher R Morgenthaler NG Bergmann A Haltmayer M Mueller T Comparative evaluation of B-type natriuretic peptide, mid-regional pro-A-type natriuretic peptide, mid-regional pro-adrenomedullin, and Copeptin to predict 1-year mortality in patients with acute destabilized heart failure J Card Fail 2007 13 42 49 10.1016/j.cardfail.2006.09.004 17339002

[B23] Dieplinger B Gegenhuber A Haltmayer M Mueller T Evaluation of novel biomarkers for the diagnosis of acute destabilised heart failure in patients with shortness of breath Heart 2009 95 1508 1513 10.1136/hrt.2009.170696 19525245

[B24] Collinson PO Barnes SC Gaze DC Galasko G Lahiri A Senior R Analytical performance of the N terminal pro B type natriuretic peptide (NT-proBNP) assay on the Elecsys 1010 and 2010 analysers Eur J Heart Fail 2004 6 365 368 10.1016/j.ejheart.2004.01.011 14987590

[B25] Mueller T Gegenhuber A Poelz W Haltmayer M Preliminary evaluation of the AxSYM B-type natriuretic peptide (BNP) assay and comparison with the ADVIA Centaur BNP assay Clin Chem 2004 50 1104 1106 10.1373/clinchem.2004.031484 15161739

[B26] Hanley JA McNeil BJ A method of comparing the areas under receiver operating characteristic curves derived from the same cases Radiology 1983 148 839 843 6878708 10.1148/radiology.148.3.6878708

[B27] Pencina MJ D'Agostino RB Sr D'Agostino RB Jr Vasan RS Evaluating the added predictive ability of a new marker: from area under the ROC curve to reclassification and beyond Stat Med 2008 27 157 172 discussion 207-112 10.1002/sim.2929 17569110

[B28] Katan M Morgenthaler N Widmer I Puder JJ Konig C Muller B Christ-Crain M Copeptin, a stable peptide derived from the vasopressin precursor, correlates with the individual stress level Neuro Endocrinol Lett 2008 29 341 346 18580851

[B29] Seligman R Papassotiriou J Morgenthaler NG Meisner M Teixeira PJ Copeptin, a novel prognostic biomarker in ventilator-associated pneumonia Crit Care 2008 12 R11 10.1186/cc6780 18252006 PMC2374597

[B30] Oghlakian G Klapholz M Vasopressin and vasopressin receptor antagonists in heart failure Cardiol Rev 2009 17 10 15 10.1097/CRD.0b013e318190e72c 19092365

[B31] Lee CR Watkins ML Patterson JH Gattis W O'Connor CM Gheorghiade M Adams KF Jr Vasopressin: a new target for the treatment of heart failure Am Heart J 2003 146 9 18 10.1016/S0002-8703(02)94708-3 12851603

[B32] Westphal M Sielenkamper AW Van Aken H Stubbe HD Daudel F Schepers R Schulte S Bone HG Dopexamine reverses the vasopressin-associated impairment in tissue oxygen supply but decreases systemic blood pressure in ovine endotoxemia Anesth Analg 2004 99 878 885 table of contents 10.1213/01.ANE.0000131970.54062.1C 15333425

[B33] Perez R Espinoza M Riquelme R Parer JT Llanos AJ Arginine vasopressin mediates cardiovascular responses to hypoxemia in fetal sheep Am J Physiol 1989 256 R1011 1018 2719144 10.1152/ajpregu.1989.256.5.R1011

[B34] Jin HK Yang RH Chen YF Thornton RM Jackson RM Oparil S Hemodynamic effects of arginine vasopressin in rats adapted to chronic hypoxia J Appl Physiol 1989 66 151 160 2917918 10.1152/jappl.1989.66.1.151

[B35] Sharples PM Seckl JR Human D Lightman SL Dunger DB Plasma and cerebrospinal fluid arginine vasopressin in patients with and without fever Arch Dis Child 1992 67 998 1002 10.1136/adc.67.8.998 1520019 PMC1793597

[B36] Januzzi JL Jr Camargo CA Anwaruddin S Baggish AL Chen AA Krauser DG Tung R Cameron R Nagurney JT Chae CU Lloyd-Jones DM Brown DF Foran-Melanson S Sluss PM Lee-Lewandrowski E Lewandrowski KB The N-terminal Pro-BNP investigation of dyspnea in the emergency department (PRIDE) study Am J Cardiol 2005 95 948 954 10.1016/j.amjcard.2004.12.032 15820160

[B37] Pratt CM Greenway PS Schoenfeld MH Hibben ML Reiffel JA Exploration of the precision of classifying sudden cardiac death. Implications for the interpretation of clinical trials Circulation 1996 93 519 524 8565170 10.1161/01.cir.93.3.519

